# Coccidioidomycosis Cases at a Regional Referral Center, West Texas, USA, 2013–2019

**DOI:** 10.3201/eid2804.211912

**Published:** 2022-04

**Authors:** Christopher Peterson, Victoria Chu, Jessica Lovelace, Mhd Hasan Almekdash, Mark Lacy

**Affiliations:** Texas Tech University Health Sciences Center School of Medicine, Lubbock, Texas, USA (C. Peterson, V. Chu, J. Lovelace);; Texas Tech University Health Sciences Center Clinical Research Institute, Lubbock (M.H. Almekdash);; University of New Mexico School of Medicine, Albuquerque, New Mexico, USA (M. Lacy)

**Keywords:** coccidioidomycosis, valley fever, occupation, demographics, Texas, fungi, United States

## Abstract

We describe 73 patients with coccidioidomycosis diagnosed or treated at a regional referral center in West Texas, USA. Patients most at risk worked in oil production or agriculture; the most-associated health factors were smoking and diabetes. Patient demographics suggest that access to care may affect coccidioidomycosis diagnosis in this region.

*Coccidioides immitis* and *C. posadasii* are soil-dwelling fungi that cause the disease coccidioidomycosis, also known as Valley fever (*1*). Since coccidiomycosis was first recorded in 1892, the disease has become a public health concern in the United States, and several thousand cases are reported annually (*2*). Severe cases may involve complicated pneumonia, musculoskeletal disease, and meningitis. *Coccidioides* arthroconidia, which reside in the soil in dry, arid climates, are endemic in the western United States, as well as in Central and South America (*2*). In West Texas, a 30-county region in western Texas, the arid climate and prevalence of at-risk occupations in oil, construction, and agricultural enterprises provide conditions for contracting the infection (*3*). However, epidemiologic and serologic studies about coccidioidomycosis in Texas are limited, and few provide data on patient risk factors, such as occupation and contributing conditions, in part because coccidioidomycosis is not a reportable disease in the state. We retrospectively examined demographics and risk factors related to coccidioidomycosis case-patients seeking treatment at a regional referral center in West Texas.

## The Study

We scanned medical records and identified patients diagnosed with coccidiomycosis during January 1, 2013–December 1, 2019, based on International Classification of Diseases, 9th Revision (ICD-9), and International Classification of Diseases, 10th Revision (ICD-10), codes 114 and B38. To be included, case-patients had to be 9–89 years of age, have a confirmed diagnosis of coccidioidomycosis, and have been diagnosed or treated at the regional referral center during the surveillance period. We separately calculated another set of demographics and risk factors at a regional hospital in another West Texas county (Appendix). We confirmed diagnoses based on provider notes or laboratory results.

We included signs and symptoms considered to be associated with coccidioidomycosis only if present at the time of diagnosis. The list of associated factors was not meant to be exhaustive, and certain risk factors (such as dust exposure) may be relevant only in areas where coccidioidomycosis is endemic. We obtained medical and social history directly from patient charts where possible. We identified at-risk occupations based on National Institute for Occupational Safety & Health status (*4*). We considered smoking status and travel history associated factors only if specified as such in case records. We defined immunocompromised status as having received chemotherapy or immunosuppressive medication within 3 months of coccidioidomycosis diagnosis, having been diagnosed with immunosuppressive disease, or having a CD4 count <200 cells/mm^3^. We defined chronic lung disease as having asthma, chronic obstructive pulmonary disorder, pneumonitis, cystic fibrosis, or other medically recognized chronic lung pathology. We defined previous lifetime exposure to an endemic site as travel to or residence in Arizona, California, New Mexico, Nevada, Utah, Mexico, or Texas (if living outside the state at time of diagnosis), all of which are coccidioidomycosis-endemic states. 

We determined coccidioidomycosis pathology on the basis of information in pathology or radiology reports or physician notes; for cause of death, coccidioidomycosis had to be specified as the cause of death in a death certificate or other medical record. We recorded any data fields for which we could not verify information from medical records as unknown. We occasionally excluded patient demographic data if records were unclear, missing, or contained contradictory information. We obtained Texas population data used in the model from the US Census Bureau (https://www.census.gov) and calculated workforce percentages using 2020 Census data. We performed statistical analysis using R software (https://www.r-project.org). We ran univariate logistic regression models to explore the association between variables of interest (sex, race and ethnicity, age, and smoking history) and outcomes (multiple risk factors, central nervous system [CNS] pathology).

We identified 73 patients with coccidioidomycosis (Table). Fluctuations in annual case totals were consistent with those previously observed in annual coccidioidomycosis counts (Figure) and were possibly related to environmental and climate factors (*5*). Among case-patients, 3 died from coccidioidomycosis. The most frequent at-risk occupations were oil and gas extraction (8/73, 11.0%) and agriculture (3/73, 4.1%), both industries common in West Texas and eastern New Mexico. Coccidioidomycosis has been associated with professions that involved dust exposure and outbreaks have been associated with exposure at job sites. One study found that more than half of the outbreaks they examined over 75 years involved occupational exposures (*6*). However, because limited reports of occupation-related cases of coccidioidomycosis exist, in part because standardized surveillance records do not include occupation, determining if the level of association is typical or accurate is difficult. Of note, Texas has the largest oil and gas workforce in the United States (334,400 workers in direct extraction and support services in 2019) (*7*) and one of the largest agricultural workforces (143,763 hired farm laborers in 2017) (*8*). The case rates we observed for the oil and gas extraction (11.0%) and agricultural (4.1%) industries were much higher than our calculated estimates for those industries in Texas, 1.15% for direct oil and gas extraction and 0.49% for agricultural farm laborers. These data support previous observations regarding these occupations as being high risk for coccidioidomycosis (*4*).

**Table:** Demographics of patients diagnosed with or treated for coccidioidomycosis at a referral center in West Texas, USA, 2013–2019

Demographics	No./total† (%)
Residence at time of diagnosis	
Texas	39/73 (53.4)
New Mexico	18/73 (24.7)
Mississippi	2/73 (2.7)
Unknown	14/73 (19.2)
Age at diagnosis, y	
<20	5/73 (6.8)
20-29	11/73 (15.1)
30-39	11/73 (15.1)
40-49	14/73 (19.2)
50-59	19/73 (26.0)
60-69	7/73 (9.6)
70-79	4/73 (5.5)
>80	1/73 (1.4)
Unknown	1/73 (1.4)
Sex	
M	56/73 (76.7)
F	17/73 (23.2)
Race and ethnicity	
Non-Hispanic Black	14/73 (19.2)
Non-Hispanic White	30/73 (41.1)
Hispanic	29/73 (39.7)
Asian/Pacific Islander	NA
Associated factors	
Smoking history	32/71 (45.1)
Diabetes	25/72 (34.7)
Immunocompromised	13/70 (18.6)
Chronic lung disease	11/71 (15.5)
Incarceration history	13 /70(18.6)
At-risk occupation	13/73 (17.8)
Endemic travel or residence‡	20/50 (40.0)
At-risk occupation	
Oil or gas extraction	8/73 (11.0)
Agriculture	3/73 (4.1)
Construction	3/73 (4.1)
Military	2/73 (2.7)
Prison worker or correctional officer	2/73 (2.7)
Truck driver	2/73 (2.7)
Mining	1/73 (1.4)
Lung pathology	
Nodule or mass	37/7 ( 52.1)
Pneumonia or consolidation	26/71 (36.6)
Cavitation	21/71 (29.6)
Effusion	14/71 (19.7)
CNS pathology	
Meningitis	8/73 (11.0)
Brain abscess or lesion	2/73 (2.7)

*CNS, central nervous system; NA, not available †N = 73 patients in study. Total <73 indicates missing information. ‡Includes only patients for whom a travel history was taken or who resided outside of Texas at time of diagnosis.

**Figure:**
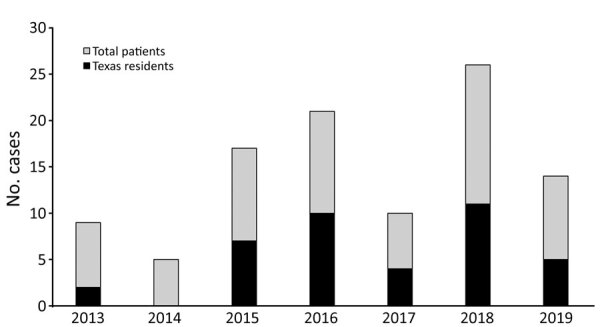
Annual cases of coccidioidomycosis for patients with a confirmed address at time of diagnosis seen at a regional referral center in West Texas, USA, 2013–2019

Most (55/73, 75.3%) patients were 20–59 years of age. Given that older patients may be more susceptible to severe illness and more likely to have comorbidities, surprisingly few (12/73, 16.4%) were >60 years of age; surveillance data have typically shown a larger proportion of patients >60 years of age (*5*). The reasons for this discrepancy are unclear but may include differences in age demographics between regions. As expected, most cases manifested with pulmonary disease, although 14% had a CNS pathology, including meningitis. A study of US Department of Veterans Affairs patients in the 1950s documented a meningitis rate of 3.5% associated with coccidiomycosis (*9*).

Of associated factors, we most frequently observed a smoking history (32/71, 45.1%) and diabetes (25/72, 34.7%). Smoking rate was consistent with the 49.1% observed in one study (*10*) and lower than the 72.0% observed in another (*11*). The frequency of diabetes we observed supports surveillance data linking diabetes and coccidioidomycosis (*12*,*13*). Thus, the association of diabetes with coccidioidomycosis may be important for populations with high diabetes prevalence and an important consideration for clinicians treating diabetic patients in *Coccidioides*–endemic regions. Smoking, sex, and race and ethnicity were not significant predictors of CNS pathology. Regional demographics, such as socioeconomic status, may play a role in access to care and diagnosis and treatment of coccidioidomycosis (*14*), especially in rural regions such as West Texas and eastern New Mexico, where patients may live several hundred miles from facilities providing advanced levels of care. Furthermore, limited access to infectious disease specialists suggests the possibility of delayed diagnoses and increased case severity in this region. Indeed, a 2017 report showed West Texas and parts of eastern New Mexico averaged <1 infectious disease physician/100,000 persons (*15*). Of the patients known to be residing in Texas at the time of diagnosis, 18/39 (46.2%) lived >50 miles from the referral center, which suggests the importance of access to higher levels of care for coccidioidomycosis diagnosis and treatment.

## Conclusions

We anticipate the need for future studies to provide a longitudinal assessment of coccidioidomycosis in Texas. Retrospective reviews from medical records, although useful, are limited in their ability to thoroughly survey the prevalence of a disease such as coccidioidomycosis within a particular region, suggesting the need for more routine surveillance such as statewide mandatory reporting. Our findings also suggest that access to higher levels of care should be considered when treating populations at risk for coccidioidomycosis. 

Appendix Additional information on coccidioidomycosis cases at a regional referral center in West Texas
