# Supplementary material for: Coccidioidomycosis Cases at a Regional Referral Center, West Texas, USA, 2013–2019
Source: Emerg Infect Dis. 2022 Apr;28(4):848–51. doi: 10.3201/eid2804.211912 (PMC8962906; doi:10.3201/eid2804.211912)

Article DOI: https://doi.org/10.3201/eid2804.211912

Coccidioidomycosis Cases at a Regional Referral Center, West Texas, USA, 2013–2019

Appendix

Using the same methods described in the main text, we collected patient information (Appendix Table) from a regional hospital located in a West Texas county different from the regional referral center providing the data examined in the main text. Differences in electronic medical record software and recordkeeping between the 2 surveyed hospitals resulted in some demographic information, such as social demographics, being significantly more limited at this hospital; some health information was unavailable because of recordkeeping limitations. We did not include missing data fields in calculations of overall totals. We collected data during the same surveillance period, January 1, 2016–December 31, 2019, because of site-specific implementation of electronic medical records.

Appendix Table. Demographics of patients diagnosed with or treated for coccidioidomycosis at a regional referral center in western Texas, 2016–2019

Demographics	No./total* (%)
Residence at diagnosis	
Texas	50/81 (61.7)
New Mexico	1/81 (1.2)
Mississippi	1/81 (1.2)
Unknown	29/81 (35.8)
Age at diagnosis, y	
<20	1/81 (1.2)
20–29	8/81(9.9)
30–39	10/81 (12.3)
40–49	19/81 (23.5)
50–59	15/81 (18.5)
60–69	13/81 (16.0)
70–79	6/81 (7.4)
≥80	0 (0)
Unknown	9/81 (11.1)
Sex	
Male	50/81 (61.7)
Female	31/81 (38.3)
Race/ethnicity	
Non-Hispanic Black	14/81 (17.3)
Non-Hispanic White	20/81 (23.8)
Hispanic	32/81 (39.5)
Asian/Pacific Islander	1/81 (1.2)
Unknown	14/81 (17.3)
Associated factors	
Smoking history	27/60 (45.8)
Diabetes	30/77 (38.9)
Immunocompromised	14/74 (18.7)
Chronic lung disease	10/77 (13.0)

Demographics	No./total* (%)
At-risk occupation	11/77 (14.3)
At-risk occupations	
Oil or gas extraction	8/77 (10.4)
Agriculture	1/77 (1.3)
Construction	1/77 (1.3)
Military	1/77 (1.3)

*N = 81 patients. Total <81 indicates missing information.

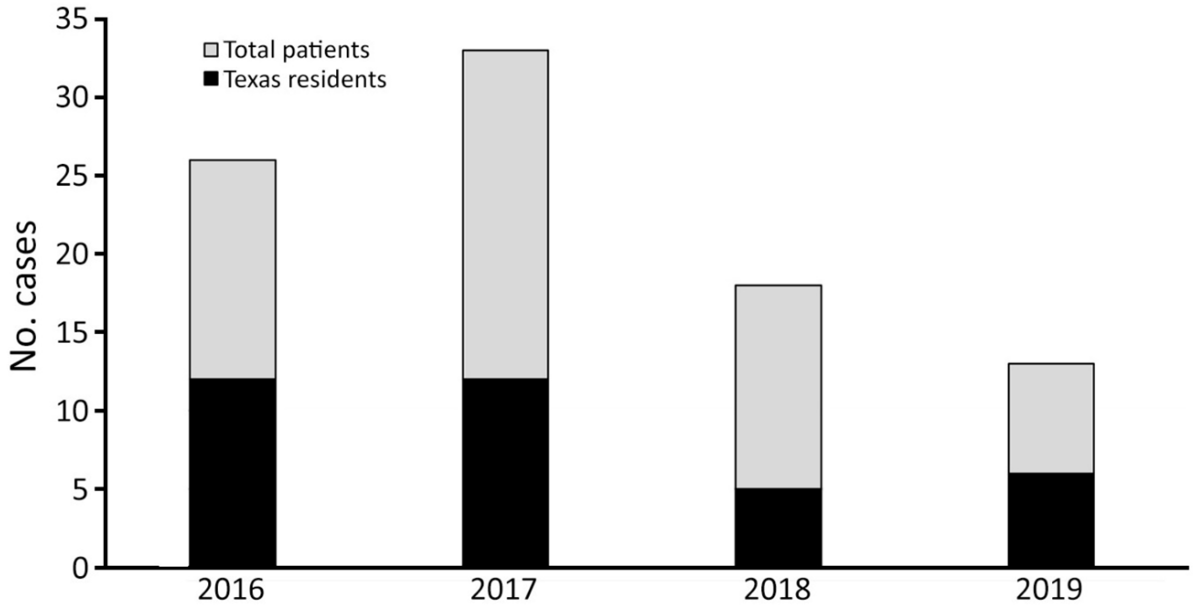

Supplement: Appendix — Additional information on coccidioidomycosis cases at a regional referral center in West Texas [file 21-1912-Techapp-s1.pdf]
